# Biomimetic Gold Nanoshell-Loaded Macrophage for Photothermal Biomedicine

**DOI:** 10.1155/2020/5869235

**Published:** 2020-04-14

**Authors:** Sung Hun Kang, Yong Kyu Lee, Il Seok Park, In-Kyu Park, Seok Min Hong, Soon Young Kwon, Young Hee Choi, Steen J. Madsen, Henry Hirschberg, Seok Jin Hong

**Affiliations:** ^1^Department of Biomedical Sciences, College of Medicine, Hallym University, Chuncheon 24252, Republic of Korea; ^2^Department of Chemical and Biological Engineering, Korea National University of Transportation, Chungju 27469, Republic of Korea; ^3^Department of Otorhinolaryngology-Head and Neck Surgery, Hallym University, Dongtan Sacred Heart Hospital, 7, Keunjaebong-gil, Hwaseong-si, Gyeonggi-do, Republic of Korea 18450; ^4^Department of Biomedical Sciences, Chonnam National University Medical School, Gwangju 61469, Republic of Korea; ^5^Department of Otorhinolaryngology-Head and Neck Surgery, Korea University College of Medicine, Ansan, Republic of Korea; ^6^Department of Pathology, Hallym University, Dongtan Sacred Heart Hospital, 7, Keunjaebong-gil, Hwaseong-si, Gyeonggi-do, Republic of Korea 18450; ^7^Department of Health Physics and Diagnostic Sciences, University of Nevada, Las Vegas 4505 S. Maryland Pkwy, Las Vegas, NV 89154-3037, USA; ^8^Beckman Laser Institute and Medical Clinic, University of California, Irvine 1002 Health Sciences Rd, Irvine, CA 92617, USA

## Abstract

The purpose of this study was to investigate the effect of photothermal treatment (PTT) with gold nanoshell (ANS) using a macrophage-mediated delivery system in a head and neck squamous cell carcinoma (HNSCC) cell line. To achieve this, ANS-loaded rat macrophages (ANS-MAs) were prepared via the coculture method with ANS. The human HNSCC (FaDu cell) and macrophage (rat macrophage; NR8383 cell) hybrid spheroid models were generated by the centrifugation method to determine the possibility of using ANS-MAs as a cancer therapy. These ANS-MAs were set into the tumor and macrophage hybrid spheroid model to measure PTT efficacy. Kinetic analysis of the spheroid growth pattern revealed that this PTT process caused a decreasing pattern in the volume of the hybrid model containing ANS-MAs (*p* < 0.001). Comparison with empty macrophages showed harmony between ANS and laser irradiation for the generation of PTT. An annexin V/dead cell marker assay indicated that the PTT-treated hybrid model induced increasing apoptosis and dead cells. Further studies on the toxicity of ANS-MAs are needed to reveal whether it can be considered biocompatible. In summary, the ANS was prepared with a macrophage as the delivery method and protective carrier. The ANS was successfully localized to the macrophages, and their photoabsorption property was stationary. This strategy showed significant growth inhibition of the tumor and macrophage spheroid model under NIR laser irradiation. In vivo toxicology results suggest that ANS-MA is a promising candidate for a biocompatible strategy to overcome the limitations of fabricated nanomaterials. This ANS-MA delivery and PTT strategy may potentially lead to improvements in the quality of life of patients with HNSCC by providing a biocompatible, minimally invasive modality for cancer treatment.

## 1. Introduction

Photo-based therapy is a newly developed therapeutic strategy with unique advantages including high specificity, minimal invasiveness, and precise spatial-temporal selectivity. Photothermal therapy (PTT), a type of photo-based therapy, has been developed for the eradication of cancer cells in the primary tumor and the initial stage of cancer metastasis. PTT can also be combined with current therapies to improve their therapeutic outcomes [1–4]. The therapeutic efficacy of PTT depends on the transformation of light to sufficient heat with photothermal agents such as metal nanostructures, nanocarbons, and organic agents [1]. Metallic nanoparticles are preferable photothermal agents due to their potential applications with tunable optical activities. Metallic nanoparticles have unique optical properties due to the interaction between light and the free conduction-band electrons on the surface of the particles. The electric field causes the collective oscillation of the conduction-band electrons on the surface of the nanoparticles under suitable light conditions. This phenomenon, termed surface plasmon resonance (SPR), makes metallic nanoparticles attractive to cancer treatment researchers [5]. Many researchers have tried to improve the inherent properties of the metallic nanoparticles by employing several strategies such as changing the nanostructure and using multiple metallic compound combinations. The combination of the Fe_7_S_8_ and Bi_2_S_3_ metallic compounds helps create a large surface area for effective drug loading and provides high NIR absorption for enhanced photothermal efficacy [3]. Thus, the combination of the two metal structures showed 1.54 times higher NIR absorption intensity than that of pure Bi_2_S_3_ nanomaterials [3]. Zhang et al. have developed the superstructure of CuS for use in chemo-photothermal therapy; this CuS was biodegradable, which enabled its use in synergistic chemo-photothermal therapy [4].

Gold-silica nanoshells, a type of gold-based nanoparticle, are excellent PTT agents due to optimum absorption, significant penetration in biological tissues under NIR light, and high optical-to-thermal energy conversion efficiency, approximately one million times higher than indocyanine green [5, 6]. The gold-silica nanoshells, first described by Oldenburg et al. [7], are obtained by using a silica core (50-500 nm) and gold layer (5-20 nm) to improve photothermal efficacy [8]. Hirsch et al. [9] demonstrated the potential of PTT by using gold-silica nanoshells with several in vitro studies on a variety of cancer cell lines, such as the human breast, prostate, brain, and liver [10].

While the gold nanoshell was the infeasible factor during target delivery to the tumor, other concerns include a vague evaluation of gold nanoshells in the toxicity study and the limitation of their efficacy in tumors [11]. The main reason for this issue is that bare nanoparticles are structurally limited by the heterogeneous tumor microenvironment, which prevents nanoparticles, including drugs, from entering deep into the extravascular tumor tissue. Malignant solid tumors consist of dense tumor cells that restrict the space for angiogenesis, resulting in tortuous and dysfunctional vasculatures that produce irregular blood supply, high interstitial fluid pressure (IFP, 10-100 mm Hg), and a compact extracellular matrix within tumor tissue [12, 13]. Moreover, the systemic administration of anticancer agents produces generalized or organ-specific toxicity in the patients. As a result, a cancer-targeted delivery system is urgently needed [11, 14].

The targeted delivery of nanoparticles is a challenging modality for bioimaging and cancer treatments. Nanoparticles may increase the efficacy of chemotherapeutics while decreasing undesired side effects. In the nanoparticle-based targeted delivery field, many researchers have tried several approaches to improve delivery efficacy, including biodegradable and biocompatible polymerization or the attachment of an antibody to a target (a specific organ or cell) [15]. However, many nanomedicine applications that are based on targeted delivery and therapy have limitations, including insufficient accumulation at the target site, uncontrolled accumulation in normal tissue, cell toxicity, and rapid elimination. To overcome these limitations, cancer therapies using immune cells have recently been proposed in which immune cells function as vectors or carriers for the delivery of therapeutic agents [6, 11, 16–18].

The biomimetic delivery system (BDS) has been emerging as a novel strategy due to its inherent tumor-homing tendency and biocompatibility [19]. The basic theory of BDS is the chemotaxis of certain cells; a variety of cytokines secreted by tumor cells attract immune cells, which accumulate in tumor sites. This strategy uses the isolated macrophages, T cells, or mesenchymal stem cells (MSCs) from peripheral blood or tumor samples, which are modified or loaded with therapeutic agents and infused back into patients to perform the treatment [17]. Recent studies also characterized immune infiltrates in the tumor microenvironment of HNSCC. This research suggests improved patient survival and high levels of intratumoral immune cell infiltrates [20]. Macrophages, which are circulating cells, are recommended by many studies for the treatment of tumors because the use of monocytes/macrophages provides several advantages compared to MSCs: they can be easily obtained from the patient, easily loaded into therapeutic drugs or nanoparticles, and reinjected into the bloodstream [5, 11, 21, 22]. Additionally, macrophages naturally penetrate tumor tissue due to the fact that tumor-associated macrophages (TAM) are one of the most prominent components of tumor mass (up to 50%) [11, 23].

In this study, the gold nanoshell-loaded macrophage (ANS-MA) system was selected as a therapeutic agent to treat HNSCC. The PEGylation of gold nanoshell (ANS) was used to increase the uptake ratio of ANS and to give the macrophage a nontoxic property [5]. The macrophage was chosen as the delivery vector to target the HNSCC, since it can provide biocompatibility and targeted delivery, and it does not degrade in the body fluid environment [24]. The HNSCC tumor cell (FaDu cell) and macrophage hybrid models were used to mimic tumor environments, such as TAM, and to determine therapeutic efficacy under the 810 nm laser light generated by PTT. This macrophage-based targeted delivery system may provide an enhanced target delivery system for cancer therapy in the medical and pharmacological fields [16–18]. The macrophage and ANS hybrid system works synergistically to optimize target delivery and improve the therapeutic outcome of HNSCC treatment.

## 2. Materials and Methods

### 2.1. Materials

**Figure 1:**
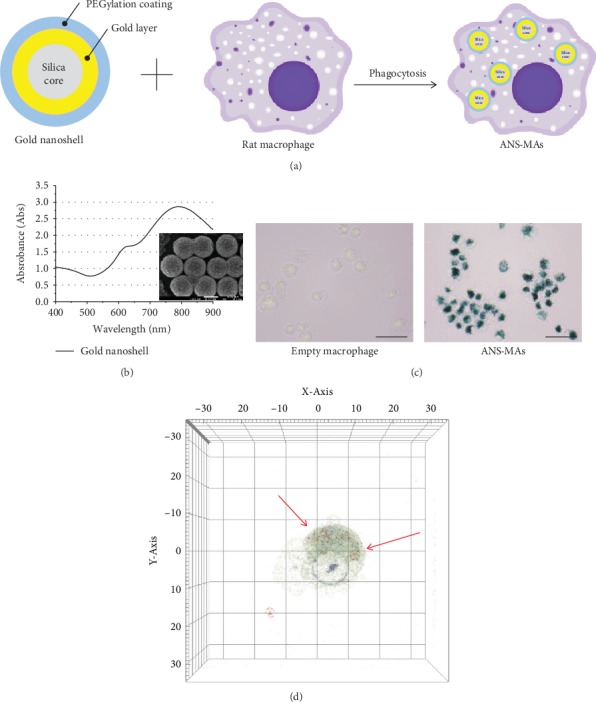
(a) Schematic image exhibits the constitution of ANS and ANS-loaded rat alveolar macrophage cells. (b) UV-visible spectrum of ANS, which exhibits a maximum absorption peak at 800–810 nm. And SEM image shows ANS size (152~162 nm) and globular morphology. (c) Phase-contrast microscopic images of empty macrophage and ANS-MAs. The macrophage cells were loaded with ANS by a coculture method for 24 hours (scale bar denotes 100 *μ*m). (d) Holography image of 3D ANS-MAs. Red dots (and red arrow) indicated the ANS location (ANS: gold nanoshell; ANS-MAs: gold nanoshell-loaded macrophages).

FaDu cells (human head and neck squamous cell carcinoma: HNSCC, ATCC, and HTB-43) were grown in Dulbecco's Modified Eagle Medium (DMEM, Gibco, Carlsbad, CA) with high glucose (Invitrogen Corp., Carlsbad, CA) supplemented with 10% fetal bovine serum (FBS, Gibco, Carlsbad, CA), 2 mM L-glutamine, and 100 mg/ml gentamycin. Rat alveolar macrophages (NR8383; ATCC, CRL-2192) were maintained in DMEM with high glucose supplemented with 10% FBS, 25 mM HEPES buffer (Gibco, Carlsbad, CA) (pH 7.4), 100 U/ml penicillin, and 100 mg/ml streptomycin. All cultures were maintained at 37°C and 7.5% CO_2_. The gold nanoshells (ANS, Nanospectra Biosciences, Inc., Houston, TX) used in this study consist of a 120 nm diameter silica core surrounded by a 12 to 15 nm thick gold shell. The nanoshells are PEGylated to inhibit aggregation and have peak absorption between 800 and 810 nm (Figure 1(b)).

### 2.2. Uptake of ANS by Macrophages

Macrophages were prepared in a 50 ml conical tube with 1 × 10^6^ cell/10 ml of culture medium concentration. After overnight incubation, macrophages were detaching to the plastic, all of the media exchanging to 10 ml of fresh culture media with 50 *μ*l of PEGylated ANS colloid (2.8 × 10^11^ particles/ml). ANS-MAs were rinsed three times by Hanks' Balanced Salt Solution (HBSS) with calcium chloride and magnesium chloride (Gibco, Carlsbad, CA) to remove excess noningested ANS. ANS-MAs were detached by using TrypLE Express (Invitrogen, Carlsbad, CA). ANS-MAs were confirmed by phase contrast microscopy and holographic images and the ANS location identified in the macrophage by the gold ion detection with scanning electron microscopy (SEM, JSM-7610F, JEOL, Akishima, Japan).

### 2.3. Photothermal Conversion Efficiency of ANS-MAs

To measure the photothermal conversion efficiency, the nanoparticle solution was placed in a 1 cm path length quartz cuvette. The temperature change in response to irradiation with an 810 nm laser was measured using a thermometer (IR meter, DH. the3017 DAIHAN Scientific, Wonju, Korea).

Photothermal conversion efficiency was calculated using the following:

(1) $$\begin{array}{c} \text{Photothermal conversion efficiency}=\frac{hA\left(T_{\max}-T_{\text{surr}}\right)-Q_{\text{dis}}}{I\left(1-10^{-A_{\lambda }}\right)}, \end{array}$$

where “*h*” indicates the heat transfer coefficient and “*A*” indicates the surface area of the container. *T*_max_ and *T*_surr_ indicate the maximum temperature of the sample (*T*_max_) and surrounding temperature (*T*_surr_), respectively. *Q*_dis_ indicates the heat dissipated from the light absorbed by the quartz cell (37.8 mW). “*I*” indicates incident laser power, and *A*_*λ*_ indicates the absorbance of ANS-MAs at 810 nm. The “*hA*” was calculated by following:

(2) $$\begin{array}{c} hA=\frac{m_{D}c_{D}}{\tau }, \end{array}$$

where *m*_*D*_ (1 g) and *c*_*D*_ (4.2 J/g°C) indicate the mass and heat capacity of deionized water and *τ* indicates the system time constant. Then, we calculated the thermal time constant using

(3) $$\begin{array}{c} t=-\tau \left(\ln\frac{\Delta T}{\Delta T_{\max}}\right), \end{array}$$

where Δ*T* indicates the temperature change, i.e., temperature difference between the sample temperature and surrounding temperature. Δ*T*_max_ indicates the maximum steady-state temperature.

### 2.4. Hybrid Tumor and Macrophage Spheroids

The tumor cell and macrophage spheroid preparation method was first described by Ivascu and Kubbies [25]. Following the described method, spheroid formation was generated by ultralow attachment surface 96-well round-bottomed plates (Corning Inc., NY) with4 × 10^3^cells per 200 *μ*l culture media in each well. Macrophages were mixed with tumor cells at ratios of tumor cell: macrophage follows 4 : 1, 5 : 1, 8 : 1, and 10 : 1. Empty or ANS-MAs were pretreated with mitomycin C to prevent cell division before mixing with tumor cells. The prepared cell mixture was centrifuged at 1,000× g for 10 min. The tumor cells and macrophages formed a disk shape immediately after centrifugation. The tumor cell and macrophage cell spheroids were subsequently maintained in a 37°C CO_2_ incubator for 48 hours to allow the cells to assume a typical three-dimensional spheroid form. And hybrid tumor and macrophage spheroid sizes were acquired at 2, 5, 8, 12, and 16 days to get a growth pattern. The hybrid spheroid volume was calculated by the following equation:

(4) $$\begin{array}{c} \text{Hybrid spheroid volume}=\frac{4}{3}\times 3.14\times {\left(\frac{\text{spheroid diameter}}{2}\right)}^{3}. \end{array}$$

### 2.5. Photothermal Treatment

Hybrid spheroids containing either empty macrophage or ANS-MAs were treated with PTT and allowed to grow in culture for 19 days to determine their viability. Hybrid spheroids were formed from a mixture of 4 × 10^3^ tumor cells and 1 × 10^3^ empty macrophage or ANS-MAs. After the 4-day incubation time, each spheroid was generated by photothermal therapy under the 810 nm laser light (Coherent Inc., Santa Clara, CA) irradiation at 14 W/cm^2^ with a 3 mm diameter laser spot for 10 min. We measured the diameter of the spheroids with a microscope and calculated the volume (volume = 4/3 × 3.14 × (spheroid diameter/2)^3^) of the spheroids for 19 days.

### 2.6. In Vitro Apoptosis Assay

*In vitro* apoptosis assay was measured using Muse® Annexin V Dead Cell Kit (Millipore Corporation, USA). The hybrid spheroids were generated in ultralow attachment surface 96-well round-bottomed plates (Corning Inc., NY) with 4 × 10^3^ cells in 200 *μ*L culture medium per well. Subsequently, 810 nm laser irradiated to the hybrid spheroid for 10 min at 14 W/cm^2^. After 24 hours of incubation time, the cells were then washed twice with PBS and incubated with 100 *μ*L of Muse® Annexin V at room temperature for 20 min. The percentage of apoptosis induction was quantified by using MUSE (Millipore Corporation, USA). The experiment was repeated three times.

### 2.7. Hemolysis Study of ANS-MAs

Red blood cells (RBCs) were obtained from 6-7-week-old SD rats purchased from Orient Bio Inc. (Seoul, South Korea). The RBCs were incubated with PBS (positive control), macrophages, ANS-MAs, and 0.03% Triton® X-100 (negative control) for 3 hours and then isolated by centrifugation (1,400 rpm, R.T.). The absorbance of free hemoglobin released in the supernatant was measured at 541 nm using UV-visible spectroscopy (OPTIZEN IV, Mecasys Co. Ltd., South Korea).

### 2.8. Complete Blood Count (CBC) and Serum Biochemistry Analysis

All animal experiments were performed under the guidelines of the Chonnam National University Medical School Research Institutional Animal Care Committee, and all the experimental protocols were approved by the committee. For the CBC and serum biochemistry analysis, ANS-MAs were intravenously injected into SD rats (6–7 weeks old). The rats (*N* = 5) were maintained under specific pathogen-free conditions, and blood samples were collected from the heart when the rats were under anesthesia at 0, 1, 7, and 21 days. Whole blood was collected in K2 EDTA 5.4 mg and SSTTM (BD Vacutainer®, Franklin Lakes, USA) tubes to prevent coagulation; 2 ml of the collected blood was used for the CBC analysis, and 3 ml of the collected blood was used for the serum biochemistry analysis. We then analyzed the total number of RBC, WBC, Hct, Hb, and platelets and determined the serologic parameters related to liver and kidney functions, including the level of various enzymes, such as albumin, ALP, ALT, AST, BUN, creatinine, globulin, total protein, T.Bil, and *γ*-GTP.

RBC stands for red blood cell, WBC for white blood cell, Hct for hematocrit, Hb for hemoglobin, ALP for alkaline phosphatase, ALT for alanine aminotransferase, BUN for blood urea nitrogen, T.Bil for total bilirubin, and *γ*-GTP for gamma-glutamyl transferase.

### 2.9. Histological Analysis

The harvested hearts, lungs, livers, and kidneys of the rats were fixed with 8% paraformaldehyde for 4 hours after washing with saline. The samples were then dehydrated and embedded in paraffin, sectioned (4 *μ*m), and stained with hematoxylin and eosin. The stained slides were examined by light microscopy with ×40 (heart) and ×100 (lung, liver, and kidney) magnifications. To determine the toxicity of the ANS-MAs, histological analysis of each organ was performed and showed whether macrophage and ANS caused tissue damage and/or any pathologic impacts such as inflammation or necrosis.

### 2.10. Statistical Analysis

Data are presented as mean ± standard deviation of results obtained from three independent trials unless otherwise indicated. Analysis of variance (ANOVA) (OriginPro8) was utilized to determine statistical significance between three or more groups, respectively. *p* values < 0.05 were considered statistically significant.

## 3. Results

### 3.1. ANS-MA Preparation

**Figure 2:**
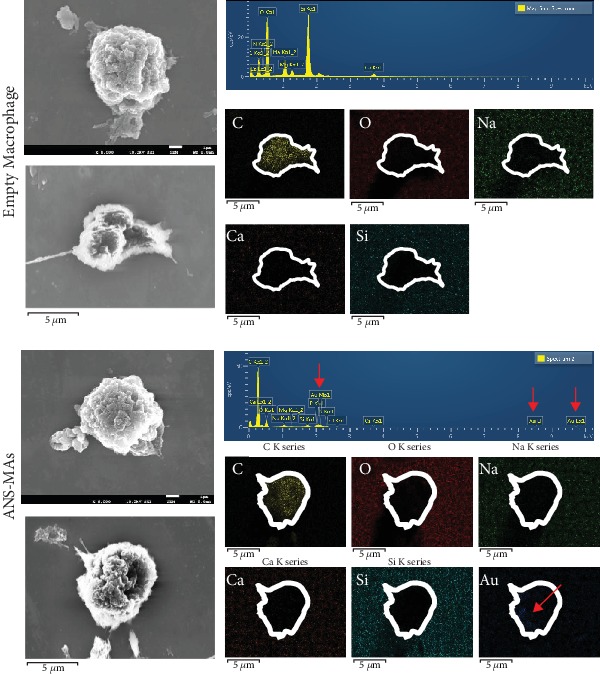
SEM image and EDX graph of the empty macrophage and ANS-MAs demonstrate that 1.39 ± 0.21 wt% Au ion was located in the macrophage cells. That Au ion means that ANS was located inside the macrophage cell. The elemental imaging process of (a) empty macrophage cells and (b) ANS-MAs indicated the Au ion site (macrophage cell shape was marked). The empty macrophage cell has no detection of the Au ions (ANS: gold nanoshell; ANS-MAs: gold nanoshell-loaded macrophages).

PEGylated ANS was characterized by UV-vis spectrometry, SEM, TEM, and X-ray diffraction (XRD) analysis. UV-vis spectra showed the maximum absorption peak at 800–810 nm for laser treatment (Figure 1(b)). SEM and TEM imaging revealed ANS size (152.0 ± 8.8 nm) and globular morphology (Figures 1(b) and S1). The structure of ANS was confirmed using XRD analysis (D2 Phaser, Bruker, Massachusetts, US). Diffraction peaks were observed at 2*θ* = 38.196, 44.395, 64.592, 77.587, and 81.744. Each peak indicated that the ANS had a structure similar to that of gold nanoparticles. In the XRD analysis, this ANS exhibited characteristic peaks at 2*θ* = 77.587 and 81.744, which correspond to those of a silica core (Figure S2). ANS-MAs (NR8383) were observed by phase-contrast microscopy and holographic analysis after a 24-hour incubation period. In Figure 1(c), the black dots indicate ANS accumulation in the macrophage. These dark areas are absent in the control macrophages that were not incubated with ANS. Holographic images (Figures 1(d) and Video 1) show ANS uptake more clearly with 3D images based on the reflective index (ANS RI: 1.334-1.342). In these images, ANS can be differentiated from other vesicles or lipid droplets. SEM and EDX images detected gold ions located in the ANS-MAs, and they show that ANS are mainly distributed in the cell cytoplasm (Figure 2). These microscopic, holographic, and EDX images indicate that the ANS were successfully inserted into the macrophage and their physical properties were safely maintained after the cell loading process. Additionally, the mass extinction coefficients for the ANS and ANS-MAs were 4.83 L(g cm)^−1^ and 4.77 L(g cm)^−1^, respectively. The similarity between the mass extinction coefficients for the ANS and ANS-MAs indicates that there is negligible interference of the macrophage caging system on the NIR absorption of the ANS.

### 3.2. Hybrid Spheroid Preparation and PTT Study

**Figure 3:**
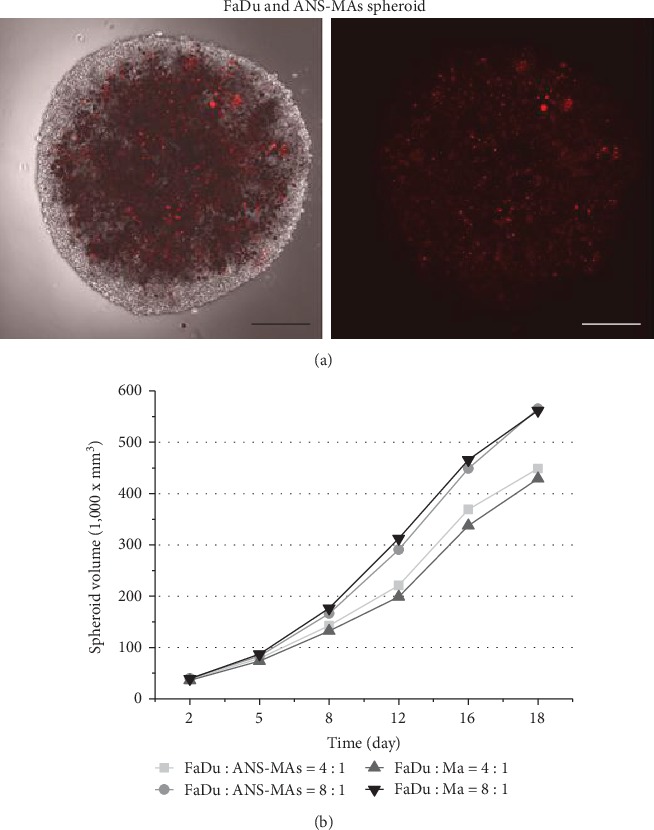
(a) Two-photon fluorescence images of FaDu and macrophage spheroid (FaDu cell: macrophage ratio = 4 : 1). Red dots mean the migration of ANS-MAs into tumor spheroids. The macrophage cell cytoplasm is stained with PKH26: red signal:. Images were acquired at a depth of approximately 50–80 *μ*m and after 48 hours incubation for spheroid formation. (scale bar denotes 5 mm). (b) The cell spheroid growth pattern detection was started after 48 hours of incubation for spheroid formation and calculated the volume of spheroid from the diameter of the spheroid. The cell spheroid growth tendency for 16 days was not changing, with or without existing ANS (ANS: gold nanoshell; ANS-MAs: gold nanoshell-loaded macrophages: Ma: macrophage).

The heat generation effect of ANS-MAs has been shown in Figure S3A. The temperature of ANS-MAs reached 51.1°C within 10 min in response to laser irradiation. The system time constant (*τ*) shown in Figure S3B was calculated using the linear fitting graph of the cooling time and temperature change data. Based on these two results, we calculated the photothermal conversion efficiency of ANS-MAs. We calculated the photothermal conversion efficiency of ANS-MAs to be 41.49%. Additionally, the photothermal effects of ANS-MAs were maintained until 5 cycles of laser treatment (Figure S3C). The hybrid spheroid model containing FaDu cells (Human HNSCC) and macrophages was produced by the centrifugation method with two ratios (FaDu : Ma = 8 : 1 and 4 : 1). ANS-MA behavior in tumor spheroids was observed via two-photon fluorescence images of cytoplasm stained with PKH26 (Figure 3(a); FaDu : Ma = 4 : 1). The growth patterns of the tumor and macrophage hybrid models, both with and without ANS applied, are shown in Figure 3(b). A slight increase from the 12.5% macrophage (FaDu : Ma = 8 : 1) to the 25% macrophage (FaDu : Ma = 4 : 1) occurred due to the different sizes between FaDu cells and macrophages. The growth ratio was not restrained with the addition of ANS.

**Figure 4:**
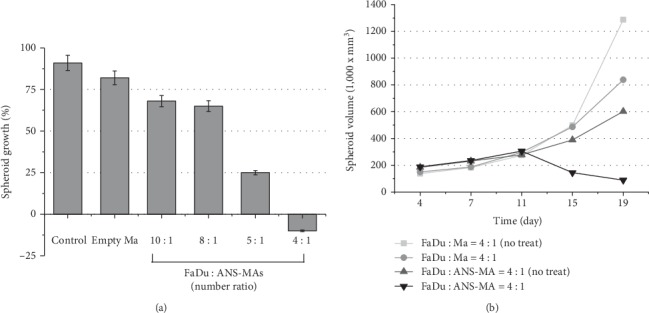
(a) The spheroid growth effect depends on the ratio of ANS-MAs with PTT. Spheroids were irradiated with 810 nm laser light for 10 min at an irradiance of 14 W/cm^2^. The values shown represent the average spheroid diameter 14 days postphotothermal treatment as a % increase of the initial size. Complete growth inhibition was observed at a FaDu : ANS-MA ratio of 4 : 1. (b) Kinetics of spheroid growth following PTT. PTT of spheroids containing ANS-MAs resulted in a decrease in the spheroid volume. In contrast, the untreated spheroids and spheroids containing empty macrophage continued to increase in volume (PTT: photothermal therapy; Ma: macrophage; ANS-MAs: gold nanoshell-loaded macrophages).

ANS-MAs induce the heat and destruction of tumor cells in the tumor and ANS-MA hybrid models. This phenomenon depends on the ratio of ANS-MAs. PTT efficacy is highest in 25% macrophages (FaDu : Ma = 4 : 1) of all the various tumor-to-macrophage ratios (FaDu control, empty Ma, FaDu : Ma = 10 : 1, 8 : 1, 5 : 1, and 4 : 1) under laser treatment (Figure 4(a)). The tumor and ANS-MA hybrid model volume growth pattern is shown in Figure 4(b). This graph demonstrates that ANS-MAs are the key factor for therapeutic agents under laser irradiation. This PTT process caused a significant decrease in the volume of the hybrid model containing ANS-MAs (*p* < 0.001). For the other groups, the ANS-MA treatment or laser treatment with empty macrophages (without ANS) was used for comparison. Those comparisons show the importance of the synergy of ANS and laser treatment and the inefficacy of either process alone.

**Figure 5:**
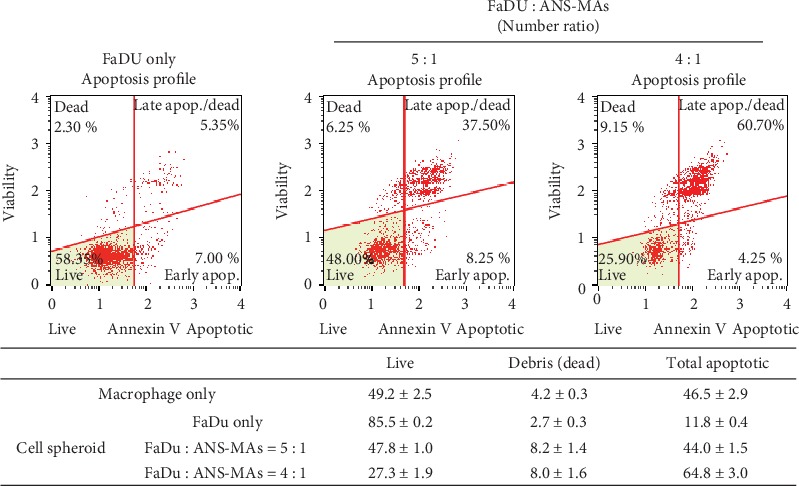
The annexin V/dead cell marker assay was performed to the induction of apoptosis and debris cell ratio in the FaDu and ANS-MAs hybrid models. Both of the models were treated with 810 nm laser light for 10 min at 14 W/cm^2^ (Ma: macrophage; ANS-MAs: gold nanoshell-loaded macrophages).

An annexin V/dead cell marker assay was performed to confirm the induction of apoptosis and debris cell ratio in the tumor and ANS-MAs hybrid models. As shown in Figure 5, the ANS-MAs exhibited increased apoptosis activation compared to the FaDu cell spheroid model. The total apoptosis results were 44.0 ± 1.5% and 64.8 ± 3.0% for cells consisting of 20% (FaDu : Ma = 5 : 1) and 25% (FaDu : Ma = 4 : 1) macrophage, respectively. Notably, the hybrid model with the ANS-MA ratio of 25% (FaDu : Ma = 4 : 1) showed an increase in the late apoptosis and death and a decrease in the early apoptosis.

### 3.3. Toxicology Study of AuNP-Loaded Macrophage Using a Balb/c Rat Model

The hemolysis of RBCs was measured after 3 hours of exposure to different macrophage concentrations with or without ANS. The absorbance of free hemoglobin released from RBCs through a PBS and Triton X-100 solution was measured using a multiscan reader as shown in Figure S4. The average hemoglobin absorbance of each ANS-MA was less than 0.1, similar to the positive control (PBS). These results indicate that ANS-MAs do not destroy RBCs and are nontoxic in vascular environments.

**Figure 6:**
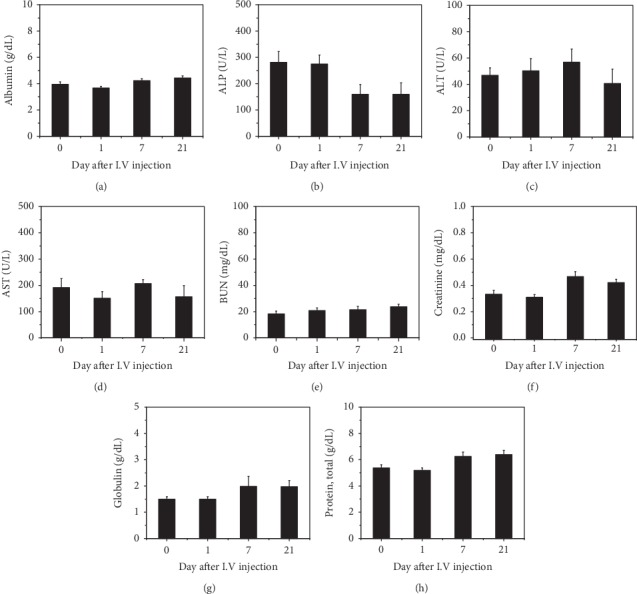
Time-dependent serum biochemistry results, from rats treated with the ANS-MAs for 21 days. Those results indicate mean of (a) albumin, (b) ALP, (c) ALT, (d) AST, (e) BUN, (f) creatinine, (g) globulin, and (h) total protein. Additionally, the values of T.Bil and *γ*-GTP were lower than the detection value (<1 and <3) (*N*-number: 7; ALP: alkaline phosphatase; ALT: alanine aminotransferase; BUN: blood urea nitrogen; T.Bil: total bilirubin; *γ*-GTP: gamma-glutamyl transferase; ANS-MAs: gold nanoshell-loaded macrophages).

**Figure 7:**
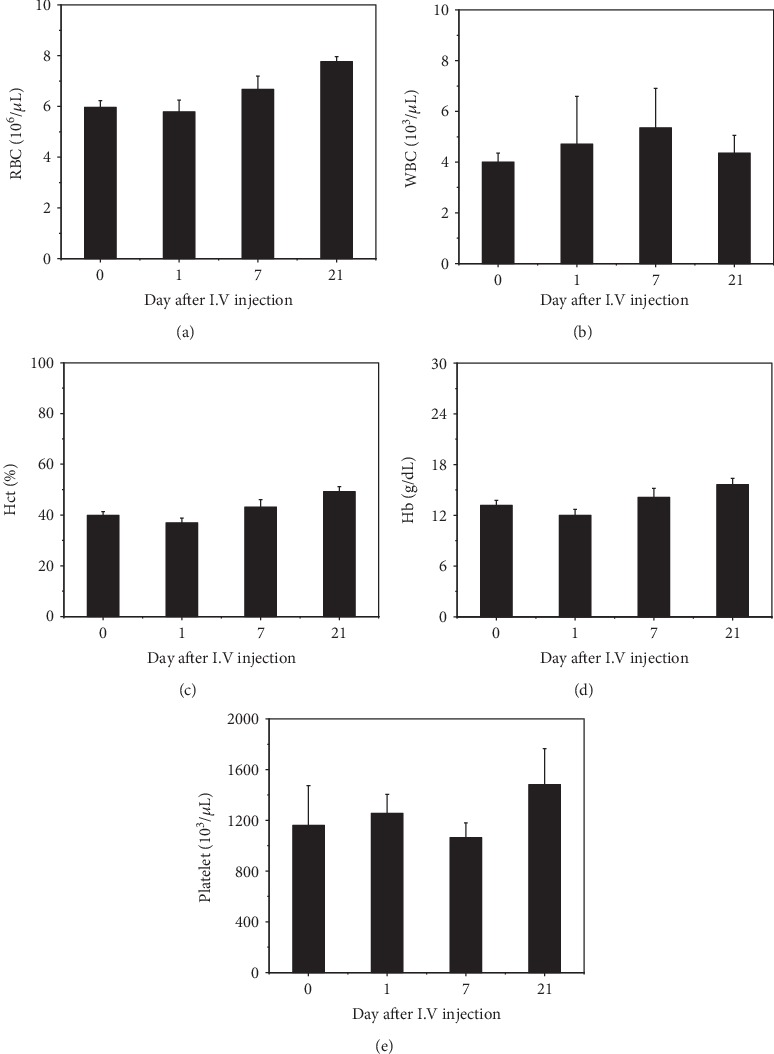
Time-dependent complete blood count test, which from rats treated with the ANS-MAs for 21 days. The values of WBC are increasing the control due to the injected macrophage cells. The values of (a) RBC, (b) WBC, (c) Hct, (d) Hb, and (e) platelets are the same with the control, keeping within the normal range. (*N*-number: 5; RBC: red blood cell; WBC: white blood cell; Hct: hematocrit; Hb: hemoglobin; ANS-MAs: gold nanoshell-loaded macrophages).

For the *in vivo* toxicology study, blood circulation half-time of ANS-MAs was calculated. The intensity of the Au ions decreased after 6 hours (maximum intensity time) postinjection, and Au ions completely become extinct at 24 hours postinjection. Based on this result, we conclude that ANS-MAs have a half-life of 12 hours in the blood. In vivo cytotoxicity studies of ANS-MAs were performed on the rat model for 21 days. The ANS-MAs were suspended in PBS and injected into the tail vein. Blood was collected from the heart before injection and at 1, 7, and 21 days postinjection. We sought to determine any toxicity, immune system alteration, or negative profiles that could result from metal- or drug-loaded macrophages as a result of their use as therapeutic or diagnostic vehicles. As shown in Figure 6, the ALP was significantly lower prior to the injection of ANS-MAs, and the ALT slowly increased until day 7, before reverting back to a normal value at day 21. The homeostasis of ALP, ALT, and AST levels was shown in our ANS-MAs, suggesting that injection may prevent liver damage or malnutrition. Blood urea nitrogen (BUN) and creatinine, which indicate healthy kidney function, were also in the normal range. Importantly, normal urea levels in the blood indicate normal kidney and liver functions. And the complete blood count (CBC) was performed at regular intervals (Figure 7). These results, including serum biochemistry and CBC, indicate normal liver function, as the values are all within normal ranges. These findings support the use of macrophages for nanoparticle or drug delivery in vivo.

**Figure 8:**
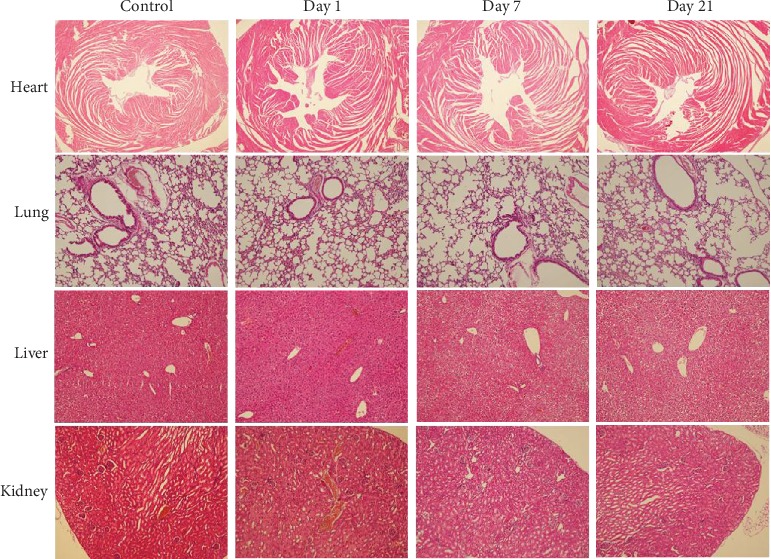
Histology study of the rats treated with the ANS-MAs for 21 days. Histological evaluation of the major organs (heart, lung, liver, and kidney) of the rat at 1, 7, and 21 days after intravenous injection of the ANS-MAs. No symptoms of inflammation and/or lesion were observed in the images. Images were taken at ×40 (heart) and ×100 (lung, liver, and kidney) magnification. (ANS-MAs: gold nanoshell-loaded macrophages).

The histological analysis, which identifies ANS-MA toxicity, was performed to detail the micromorphology and histological evaluation of tissue interactions.  Figure 8 provides a visual observation of inflammation and histological interactions in ANS-MAs. The rat heart tissue shows no significant appearance of hyperemia in the mesenchyme, with no infiltration of inflammatory corpuscles including lymphocytes, neutrophilic granulocytes, or eosinophil granulocytes. Infiltration of the inflammatory signs was not detected in the lung tissue image. The image of liver tissue was normal with hepatic lobules intact. Additionally, the symptoms of swelling, degeneration, necrosis, and infiltration of inflammatory corpuscles were not detected in the liver. In the kidneys, the size of the renal glomerulus was normal, and no inflammatory corpuscles or urinary cylinders were found. In summary, no apparent histopathological abnormalities, lesions, or necroses were observed in the heart, lungs, liver, or kidneys during the 21-day period.

## 4. Discussion

Photothermal therapy is based on the resonant photon energy absorbed with light irradiation, resulting in the rapid generation of heat on the surface of the nanoparticles, which damages and destroys cancer cells. The therapeutic strategy of PTT with nanoparticles provides localized, targeted heating and cytotoxic treatment which can potentially offer more functional and aesthetic results [2, 26, 27]. ANS have an absorption in the NIR region; however, most biological tissues lack NIR-absorbing chemophores [10, 26]. Therefore, normal tissue around the tumor might suffer minimal thermal damage. In the case of an ANS having a maximum absorption peak at 800-810 nm (Figure 1(b)), which falls within the first NIR window (650–850 nm), it can safely penetrate 2-3 cm of the tissue [28]. This penetration depth was hard to apply to the internal organs (including the brain, heart, liver, kidney, and spleen), but HNSCC is a suitable type due to the shallow penetration depth of light [29, 30].

ANS have been the subject of intense scrutiny due to their potential PTT application. However, the clinical use of nanoparticles for HNSCC treatment requires specific targeted delivery and biocompatibility. Recent research has focused on the macrophage as a delivery vessel for the targeted nanomedicine approach in cancer therapy, as it may provide enhanced therapeutic efficiency and reduced toxicity. The macrophage can cause migration and reaction in tumor development [11]. Additionally, the macrophage phagocytoses the ANS. The strategy of implementing a biomimetic macrophage delivery system would provide a nontoxic option that yields a high uptake ratio of nanoparticles [21]. In this study, ANS was PEGylated to increase the biocompatibility of gold, and a sufficient quantity of ANS was taken up by the macrophage to provide efficient PTT. Phagocytosis of the ANS was observed when incubated with the macrophage for 24 hours. The ANS locations were indicated by their optical, reflective index, and gold ion (Figures 1 and 2, and S1 and Video 1). Another cell-based delivery modality, cell-membrane-coating nanotechnology, has been widely applied in PTT [31]. Even though it has a remarkable ability to interact with its biological environment, cell-membrane-coating nanotechnology had some limitations as it can disrupt the structure of the cell membrane [31, 32]. This method involved complicated manufacturing steps. Additionally, nanomaterials used in cell-membrane-coating technology had uncontrolled membrane structure, and this method had low nanoparticle uptake ratio. Therefore, in order to circumvent the issues associated with cell-membrane-coating technique and to ensure certain advantages, we used live macrophages in this study. ANS-MAs were biocompatible, easy to prepare by coculturing macrophages with nanoparticles, and had high nanoparticle absorption. Additionally, the migration properties of ANS-MAs were similar to those of live macrophages [31]. One important feature of hyperthermia with PTT is the hypoxic cell distribution in the center of tumors. It is susceptible to hyperthermia death due to its oxygen-independent condition. This hyperthermia induces the necrosis of cancer cells with protein denaturation [2, 28, 33]. At temperatures greater than 43°C, protein denaturation and disruption of the cellular membrane occur, and some studies have shown ablation of tumor tissues [29, 34, 35]. Cytotoxic effects have been demonstrated in cells maintained at 42°C for 1 h, and irreversible cellular damage occurs when temperatures are increased to 46°C for 1 h [36]. This duration can be shortened to only 4-6 min using higher temperatures of 50-52°C [36, 37]. Yang et al. have reported that thermally induced injury and death of cancer cells begin at 44°C to 45°C, which was achieved using the PTT effect with gold NS and irradiation with an NIR laser at a power of 2 W for 5 min [38, 39]. It can be expected, then, that the PTT strategy using the ANS-MAs can induce the apoptosis and cell death progress. As the results of this study show, apoptosis and death values increased in correlation with the ANS-MA ratio (Figure 5).

Moreover, the biocompatibility of the ANS-MA system is an important factor in evaluating the therapeutic potential of the new drug-delivery system. Because biocompatibility is a critical property for blood-contacting materials, it must be examined prior to clinical use [40]. In recent decades, a majority of nanotoxicity research has focused on cell-based examination. However, these analysis studies can be misleading, so they require verification with *in vivo* animal experiments [41]. Several studies on the biodistribution of gold nanoparticles have shown that most gold nanoparticles predominantly accumulate in organs such as the liver and lung after intravenous administration. These studies revealed that the distribution of gold nanoparticles was size dependent [42, 43]. We traced gold nanoparticle-loaded macrophages in a mouse model. The results indicated that macrophages accumulated significantly in the lungs and liver, from 1 to 6 hours after intravenous administration of ANS-MAs, and were removed from each organ at 24 hours (data not shown). *In vivo* toxicology studies have been established to confirm the biocompatibility property of ANS-MAs. The hematology test was used to indicate the hemocompatibility of the system by determining whether or not a hemolytic reaction was induced. The hemolytic reaction can be caused by immune hemolysis and/or nonimmune hemolysis. According to the guide for the evaluation of biocompatible materials, the hemolysis rate should be less than 5% [40]. In the serum biochemistry, the six important factors that indicate hepatic function include albumin, globulin, glutamic pyruvate transaminase (GTP), alkaline phosphatase (ALP), total bilirubin (TB), and total protein. Normal values for each of these indicate no damage to the liver (Figure 6) [41]. A complete blood count (CBC) was performed at regular intervals, and the results suggest no acute toxicity (Figure 7) [44]. And the histological studies suggest that ANS-MAs do not burden the heart, lungs, liver, or kidneys, organs to which damage can induce toxic responses (Figure 8) [40]. In other animal model toxicology studies of intravenously injected silica nanoparticles, hepatocyte necrosis and mononuclear inflammation at the portal area were observed [45], indicating the possibility of liver toxicity due to silica nanoparticles. Metal-based photothermal nanoagents, such as CuS, had some limitations associated with toxicity [31, 46]. Feng et al. [31] have suggested that CuS-based nanoparticles can serve as promising photothermal agents; however, their biodistribution study showed that intravenously administrated CuS nanomaterials were mainly present in the spleen, liver, and lung, organs that are responsible for eliminating pathogens via the reticuloendothelial system (RES). These results suggest that CuS can potentially show organ toxicity. Our findings provide substantial evidence for the safety of ANS-MAs for biomedical applications. Our results show that ANS-MAs can be regarded as biologically safe. Furthermore, no acute toxicity or morphological changes were noted from our cytotoxicity studies as determined by the hematology test, *in vivo* serum biochemistry, CBC, and histological studies.

In this study, ANS, which can maximize the hyperthermia in PTT, were used as part of a PTT strategy to treat the HNSCC. To determine the possibility of using ANS-MAs as a cancer therapy, we prepared the ANS-MAs by rat macrophage (NR8383, Figure 1(a)). These ANS-MAs were set into tumor and macrophage spheroid models to measure PTT efficacy. The efficacy of PTT depends on a number of factors, including ANS concentration in the target tissue, laser power density, exposure time, and blood perfusion. In the present study, significant spheroid growth inhibition occurred in hybrid spheroids of tumor and ANS-MAs compared with spheroids containing empty macrophages. The dependence of PTT efficacy on the number of loaded macrophages in hybrid spheroids was investigated, and significant growth suppression was observed in spheroids consisting of 20% loaded macrophage, whereas complete growth cessation was observed at a concentration of 25% loaded macrophage (Figure 4). The cell hybrid spheroid model closely resembles the real cancer environment and provides precise measurements of therapeutic potential of our system. Toxicology studies predict that nanotechnology-mediated immune cell systems can be applied to novel strategies to overcome the limitations of various fabricated materials. As the present research shows, this macrophage-based delivery and protection strategy provides enhanced biocompatibility and a minimally invasive modality for cancer therapy in the medical and pharmacological fields.

## 5. Conclusion

This ANS-MA system is able to be prepared easily, protects the therapeutic agents from the blood immune system, and enhances the effect of PTT on HNSCC. In this study, ANS were successfully localized in the macrophage, and their physical and photoabsorption properties were steadfast. The tumor and macrophage hybrid model analysis indicated powerful ANS-based PTT efficacy under NIR laser irradiation. *In vivo* toxicology results suggest that ANS-MA could be a promising candidate for a biocompatible strategy to overcome the limitations of fabricated nanomaterials. This ANS-MA delivery and PTT system strategy may work synergistically with other therapy strategies, and it may potentially improve the quality of life of patients with HNSCC by providing a minimally invasive modality for cancer treatment.

## Data Availability

The data used to support the findings of this study are available from the corresponding author upon request.

## Supplementary Materials

Supplementary 1 Figure S1: SEM and TEM images of the ANS, which exhibit a globular morphology and sizes in the rage of 152–162 nm (ANS: gold nanoshell). Figure S2: the XRD pattern of the ANS and AuNP (with ANS structure) (ANS: gold nanoshell; AuNP: gold nanoparticles). Figure S3: (A) the temperature variation graph of the photothermal efficiency of ANS-MAs. Temperature change was induced by irradiating the ANS-MAs using an 810 nm laser. Then, the ANS-MAs were allowed to slowly cool down to surrounding temperature (30°C) after turning off the laser. (B) Linear fitting graph of the cooling time and temperature change data. The photothermal conversion efficiency of ANS-MAs was calculated using these data and graphs. (C) The temperature change graph of ANS-MAs indicated that the photothermal effect is stable for 5 cycles. The cycle involved irradiation for 10 min and blackout 10 min (total 5 times). Figure S4: hemoglobin release profile from the RBCs, which are exposed to ANS-MAs. The value was measured from the absorbance parameter of the RBC supernatant. PBS and Triton X-100 were used to each negative and positive control, respectively (RBC: red blood cell; ANS: gold nanoshell; ANS-MAs: gold nanoshell-loaded macrophages). ^∗^a: PBS (positive control); b: macrophage; c-g: ANS-MAs (c–g: 12.5, 5, 2.5, 1.25, and 0.635 pmol of ANS/cells); h: Triton X-100 (negative control).

Supplementary 2 Video 1: holography image video of 3D ANS-MAs. Red dots indicated the ANS location (ANS: gold nanoshell; ANS-MAs: gold nanoshell-loaded macrophages).
