# Supplementary material for: Biomimetic Gold Nanoshell-Loaded Macrophage for Photothermal Biomedicine
Source: Biomed Res Int. 2020 Apr 14;2020:5869235. doi: 10.1155/2020/5869235 (PMC7178525; doi:10.1155/2020/5869235)
Supplement: Supplementary 1 — Figure S1: SEM and TEM images of the ANS, which exhibit a globular morphology and sizes in the rage of 152–162 nm (ANS: gold nanoshell). Figure S2: the XRD pattern of the ANS and AuNP (with ANS structure) (ANS: gold nanoshell; AuNP: gold nanoparticles). Figure S3: (A) the temperature variation graph of the photothermal efficiency of ANS-MAs. Temperature change was induced by irradiating the ANS-MAs using an 810 nm laser. Then, the ANS-MAs were allowed to slowly cool down to surrounding temperature (30°C) after turning off the laser. (B) Linear fitting graph of the cooling time and temperature change data. The photothermal conversion efficiency of ANS-MAs was calculated using these data and graphs. (C) The temperature change graph of ANS-MAs indicated that the photothermal effect is stable for 5 cycles. The cycle involved irradiation for 10 min and blackout 10 min (total 5 times). Figure S4: hemoglobin release profile from the RBCs, which are exposed to ANS-MAs. The value was measured from the absorbance parameter of the RBC supernatant. PBS and Triton X-100 were used to each negative and positive control, respectively (RBC: red blood cell; ANS: gold nanoshell; ANS-MAs: gold nanoshell-loaded macrophages). ∗a: PBS (positive control); b: macrophage; c-g: ANS-MAs (c–g: 12.5, 5, 2.5, 1.25, and 0.635 pmol of ANS/cells); h: Triton X-100 (negative control). [file 5869235.f1.docx]

**Supporting information**

**Biomimetic Gold Nanoshells loaded Macrophage for Photothermal Biomedicine**

Sung Hun Kang^1†^, Yong Kyu Lee^2†^, Il Seok Park^3^, In-Kyu Park^4^, Seok Min Hong^3^, Soon Young Kwon^5^, Young Hee Choi^6^, Steen J. Madsen^7^, Henry Hirschberg^8^, and Seok Jin Hong^3*^

^1^ Department of Biomedical Sciences, College of Medicine, Hallym University, Chuncheon 24252, Republic of Korea

^2^ Department of Chemical and Biological Engineering, Korea National University of Transportation, Chungju 27469, Republic of Korea

^3^ Department of Otorhinolaryngology-Head and Neck Surgery, Hallym University, Dongtan Sacred Heart Hospital, 7, Keunjaebong-gil, Hwaseong-si, Gyeonggi-do, Republic of Korea, 18450

^4^ Department of Biomedical Sciences, Chonnam National University Medical School, Gwangju 61469, Republic of Korea

^5^ Department of Otorhinolaryngology-Head and Neck Surgery, Korea University College of Medicine, Ansan, Korea

^6^ Department of pathology, Hallym University, Dongtan Sacred Heart Hospital, 7, Keunjaebong-gil, Hwaseong-si, Gyeonggi-do, Republic of Korea, 18450

^7^ Department of Health physics and Diagnostic Sciences, University of Nevada, Las Vegas 4505 S. Maryland Pkwy, Las Vegas, NV, 89154-3037, United States

^8^ Beckman Laser Institute and Medical Clinic, University of Califonia, Irvine 1002 Health Sciences Rd, Irvine, CA, 92617, United States

† These authors contributed equally.

* Correspondence: Seok Jin Hong [enthsj@hanmail.net](mailto:enthsj@hanmail.net) Tel.: +82-31-8086-2670

**Figure S1.** SEM and TEM images of the ANS, which exhibit a globular morphology and sizes in the rage of 152‒162 nm. (ANS: gold nanoshell)

**Figure S2.** The XRD pattern of the ANS and AuNP (with ANS structure). (ANS: gold nanoshell, AuNP: gold nanoparticles)

**Figure S3.** (A) The temperature variation graph of the photothermal efficiency of ANS-MAs. Temperature change was induced by irradiating the ANS-MAs using an 810 nm laser. Then, the ANS-MAs were allowed to slowly cool down to surrounding temperature (30 ^o^C) after turning off the laser. (B) linear fitting graph of the cooling time and temperature change data. The photothermal conversion efficiency of ANS-MAs was calculated using these data and graphs. (C) The temperature change graph of ANS-MAs indicated that the photothermal effect is stable for 5 cycles. The cycle involved irradiation for 10 min and blackout 10 min (total 5 times).

**Figure S4.** Hemoglobin release profile from the RBCs, which exposed to ANS-MAs. The value was measured from the absorbance parameter of the RBCs supernatant. PBS and Triton X-100 were used to each negative and positive control, respectively. (RBC: Red Blood Cell, ANS: gold nanoshell, ANS-MAs: gold nanoshell loaded macrophages)

* a : PBS (Positive control) , b : Macrophage , c-g : ANS-MAs (c – g : 12.5, 5, 2.5, 1.25, and 0.635 pmol of ANS/cells) , h : Triton X-100 (Negative control)
